# Internalization of B Cell Receptors in Human EU12 **μ**HC^+^ Immature B Cells Specifically Alters Downstream Signaling Events

**DOI:** 10.1155/2013/807240

**Published:** 2013-10-09

**Authors:** Jing Liu, Wanqin Xie, Miles D. Lange, Sang Yong Hong, Kaihong Su, Zhixin Zhang

**Affiliations:** ^1^Department of Pathology and Microbiology, University of Nebraska Medical Center, LTC 11706A, Omaha, NE 68198-7660, USA; ^2^Department of Microbiology, University of Alabama at Birmingham, Birmingham, AL 35294, USA; ^3^Department of Internal Medicine, University of Nebraska Medical Center, LTC 11706A, Omaha, NE 68198-7660, USA; ^4^Eppley Institute for Research in Cancer and Allied Diseases, University of Nebraska Medical Center, LTC 11706A, Omaha, NE 68198-7660, USA

## Abstract

It has been recognized for a long time that engagement of B cell antigen receptors (BCRs) on immature B cells or mature B cells leads to completely opposite cell fate decisions. The underlying mechanism remains unclear. Here, we show that crosslinking of BCRs on human EU12 **μ**HC^+^ immature B cells resulted in complete internalization of cell surface BCRs. After loss of cell surface BCRs, restimulation of EU12 **μ**HC^+^ cells showed impaired Ca^2+^ flux, delayed SYK phosphorylation, and decreased CD19 and FOXO1 phosphorylation, which differ from those in mature Daudi or Ramos B cells with partial internalization of BCRs. In contrast, sustained phosphorylation and reactivation of ERK upon restimulation were observed in the EU12 **μ**HC^+^ cells after BCR internalization. Taken together, these results show that complete internalization of cell surface BCRs in EU12 **μ**HC^+^ cells specifically alters the downstream signaling events, which may favor receptor editing versus cell activation.

## 1. Introduction

The development of B lymphocytes from hematopoietic progenitor cells in the bone marrow (BM) into mature, circulating B cells can be divided into distinct stages according to the expression of specific cell surface markers as well as the rearrangement status of the IgH and L chain gene loci [1, 2]. Immature B cells are the first to express the complete form of the B cell antigen receptors (BCRs), which can recognize specific antigens [3]. The BCRs expressed on immature and mature B cells are all composed of membrane bound IgH/IgL peptides with noncovalently linked signaling components, the Ig*α*/Ig*β* heterodimers [4, 5]. Although the BCRs expressed on the immature and the mature B cells share the same components, stimulation through BCRs results in totally opposite outcomes. In mature B cells, BCR stimulation induces cell activation and proliferation, antibody affinity maturation, and class-switch recombination during T cell-dependent responses and ultimately drives B cells differentiating into memory B cells or antibody-secreting plasma cells [5–7]. In contrast, stimulation of immature B cells via BCRs leads to receptor editing or clonal deletion [8–11].

Most of our current understandings of BCR-mediated signaling events are derived from analyses of mature B cells. One of the early signal events following BCR crosslinking is protein tyrosine kinases (PTKs) Lyn and Syk-mediated phosphorylation of the tyrosine residues in the two immunoreceptor tyrosine-based activation motifs (ITAMs) in Ig*α* and Ig*β* [5, 12, 13]. Lyn predominantly phosphorylates the first ITAM tyrosine, whereas Syk phosphorylates and binds to both ITAM tyrosine residues [14]. The adaptor protein SH2 domain-containing leukocyte protein of 65 kDa (BLNK) is then recruited to the BCR complex, where it is phosphorylated by Syk [15]. Tyrosine phosphorylated BLNK serves as an adaptor to organize the signalosome by recruiting Btk, Vav, PLC*γ*2, Grab2, and Rac1 [16]. Formation of this signalosome is important for full induction of Ca^++^ influx and activation of the downstream PI3K, MAPK, and NF-*κ*B signaling pathways [17, 18].

Human EU12 *μ*HC^+^ cells have features of BM immature B cells [19–21]. Our previous studies have shown that *V*_*H*_ replacement occurs spontaneously in the EU12 *μ*HC^+^ cells [20]. Moreover, crosslinking of BCRs results in arrest of cell proliferation and induction of *V*_*H*_ replacement [20, 21]. Thus, the EU12 *μ*HC^+^ cells were used as an experimental model to dissect the BCR-mediated signaling events in immature B cells versus those in mature B cells. We noticed that treatment of EU12 *μ*HC^+^ cells with F(ab′)_2_ anti-IgM antibodies resulted in complete internalization of cell surface BCRs, which is in sharp contrast to the partial internalization of BCRs observed in Daudi or Ramos mature B cells. Detailed analyses showed that complete BCR internalization specifically changed BCR-mediated downstream signaling events in the EU12 *μ*HC^+^ immature B cells with attenuated Ca^++^ influx and CD19 and FOXO1 phosphorylation, but with sustained ERK activation. Such changes may favor the induction of receptor editing rather than cell proliferation in these cells.

## 2. Materials and Methods

### 2.1. Cell Lines and Treatments

The EU12 *μ*HC^+^ cells are purified by flow cytometric sorting from the parental EU12 cells, which were established from an acute lymphocytic childhood leukemia patient [20, 21]. Ramos and Daudi are B cell lymphomas obtained by tissue culture adaptation of Burkitt's lymphomas [22–24]. In all experiments, cells were cultured in RPMI 1640 medium (Invitrogen, Carlsbad, CA), supplemented with 10% heat-inactivated FBS, 100 units/mL penicillin/streptomycin, 50 *μ*M *β*-mercaptoethanol, and 2 mM L-glutamine. Cells were maintained at 0.1 to 1 × 10^6^ cells per mL. For direct stimulation, cells were stimulated with goat F(ab′)_2_ fragments of anti-human IgM antibodies (10 *μ*g/mL) (Southern Biotech, Birmingham, AL) at room temperature. For restimulation experiments, cells were pretreated with goat F(ab′)_2_ anti-human IgM antibody fragments at 2 *μ*g/mL for 30 min; then, the cells are washed with fresh tissue culture medium to remove excessive anti-IgM antibodies, recovered in complete growth medium for 2 or 4 h, and then restimulated with 10 *μ*g/mL goat F(ab′)_2 _anti-human IgM antibody or the monoclonal anti-human IgM antibody (BD Biosciences). Ca^++^ influx, Western blot, and immunofluorescence analyses were all performed under these two conditions.

### 2.2. Antibodies and Reagents

Immunofluorescence assays were performed using the following antibodies: Cy5-labeled AffiniPure donkey anti-human IgM F(ab′)_2_ fragment (Jackson ImmunoResearch, West Grove, PA), goat anti-human IgM (*μ* chain specific, LE/AF) F(ab′)_2_ fragments (Southern Biotech, Birmingham, AL), goat anti-rabbit Alexa-488 (Invitrogen, Carlsbad, CA), anti-phospho-SYK, phospho-ERK, and phospho-CD19 antibodies (Cell Signaling Technology, Danvers, MA).

### 2.3. Cell Surface Staining and Flow Cytometric Analysis

For cell surface staining, cells (1 × 10^6^ cells) were blocked with PBS buffer with 2% FBS and stained with Cy5-labeled anti-human IgM F(ab′)_2_ antibodies, FITC-labeled anti-HLA antibodies, or APC-labeled anti-CD86 antibodies on ice for 30 min [21]. After washing with PBS buffer with 2% FBS, samples were analyzed on an Accuri cytometric analyzer. Dead cells were excluded by PI staining. The results were then analyzed using the CFlow Plus software (Accuri Cytometers Inc., MI).

### 2.4. Ca^2+^ Influx Assays

Intracellular Ca^2+^ levels were monitored by flow cytometric analyses using the Fluo-3AM fluorescence dye (Molecular Probes, Eugene, OR) [21]. Briefly, EU12 *μ*HC^+^ cells (10^6^ cells/mL) were preloaded with 1 *μ*M Fluo-3AM with pluronic-F127 at 1 : 1 ratio for 30 min at 37°C in the dark. Cells were washed and resuspended in RPMI media containing 1% FBS. Cells were maintained in the dark at room temperature before use. Samples were analyzed on an Accuri cell analyzer. Unstimulated cells were used to establish the baseline of fluorescence. Different stimuli were added at 30 sec after running the samples on the Accuri cell analyzer and continued for 4 min. Flow cytometry data were analyzed using the CFlow Plus software (Accuri Cytometers Inc., MI).

### 2.5. Anti-IgM Antibody-Mediated BCR Internalization

Human EU12 *μ*HC^+^, Daudi, and Ramos cells or murine 70Z/3 cells were used in these experiments. Approximately 1 × 10^6^ cells were incubated with goat anti-human IgM antibody F(ab′)_2_ fragments (2 *μ*g/mL) for different human cell lines or goat anti-mouse IgM antibody F(ab′)_2_ fragments (5 *μ*g/mL) (Southern Biotech, AL) at 37°C. Cells were collected at different time points and cell surface BCR levels were analyzed by flow cytometry.

### 2.6. Immunofluorescence Analysis

Different cells were directly stimulated or restimulated as described above. After treatment, cells were fixed with 3.7% formaldehyde at room temperature for 10 min in the dark, followed by incubation with 0.1% Triton X-100 for 5 min on ice. Untreated cells were used for both negative and secondary antibody staining controls. Cells were washed twice with 1 x PBS and blocked with PBS + 1% goat serum at room temperature for an hour. Anti-phospho-SYK or anti-phospho-ERK antibodies (1 : 200 dilution in PBS + 2% BSA) were added to the samples and incubated at room temperature for 1 h in the dark. After extensive washing, bound antibodies were detected with goat anti-rabbit IgG-Alxa488 secondary antibody (1 : 1000 dilution) (Invitrogen, Carlsbad, CA) and visualized under an Olympus X81 fluorescence microscope. Images were captured with 60X lens under oil and analyzed using the Slidebook 5 software. 

### 2.7. Western Blot Analysis

For analysis of global protein tyrosine phosphorylation, EU12 *μ*HC^+^, Daudi, and Ramos cells (10 × 10^6^/mL) were either directly stimulated with anti-human IgM F(ab′)_2_ antibody fragments (10 *μ*g/mL) for 3 min or pretreated with goat anti-human IgM F(ab′)_2_ antibody fragments (2 *μ*g/mL) for 30 min, washed, recovered in complete RPMI medium for 4 h, and then restimulated with goat anti-human IgM antibody F(ab′)_2_ fragments (10 *μ*g/mL) for 3 min. Cells were washed once with 1 x PBS; whole cell lysates were prepared using 1% TNT buffer [21, 25]. Samples were separated onto 10% SDS-PAGE. The phosphorylation status of the BCR signaling molecules CD19, LYN, SYK, BLNK, ERK, AKT, and FOXO1 was then analyzed by Western blot with phosphospecific antibodies (Cell Signaling Technology, Danvers, MA).

## 3. Results

### 3.1. Crosslinking BCRs Induces Different Levels of BCR Internalization in Human Immature versus Mature B Cells

**Figure 1:**
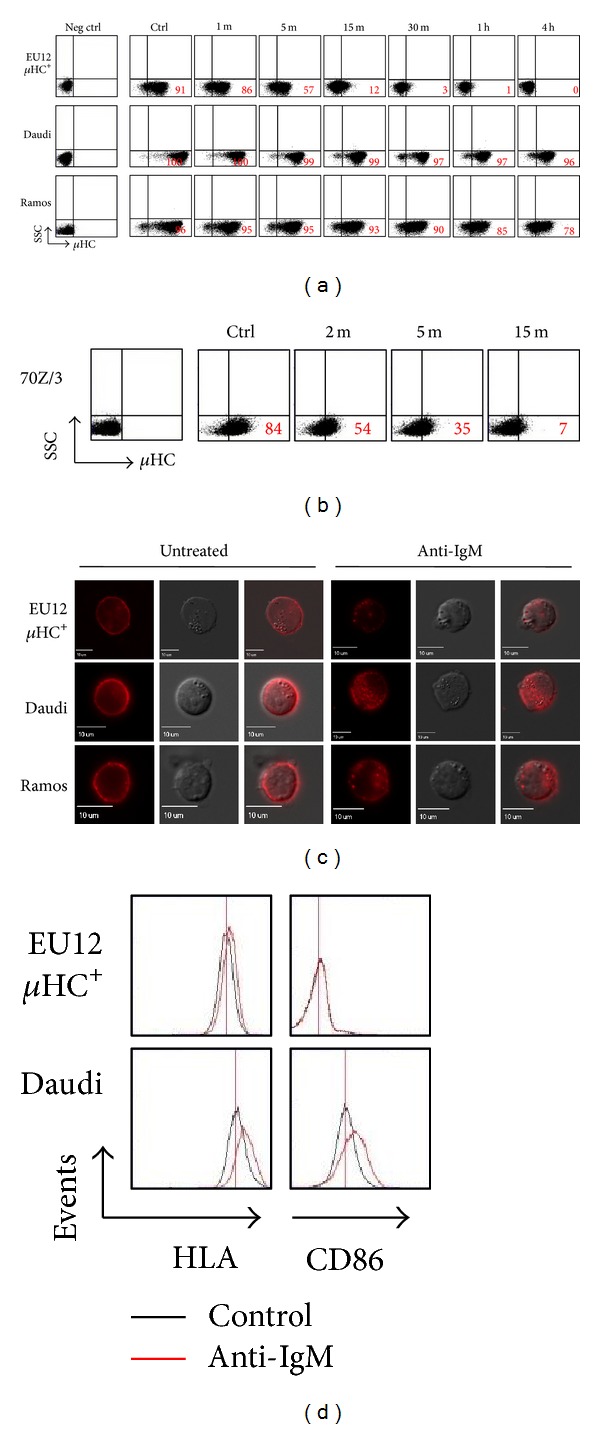
Anti-IgM antibody induces BCR internalization in EU12 *μ*HC^+^ immature B cells and in mature Daudi or Ramos B cells. (a) Human U12 *μ*HC^+^, Daudi, and Ramos cells were treated with goat anti-IgM antibody F(ab′)_2_ fragments (2 *μ*g/mL) for different times. The cells were stained with the Cy5 labeled anti-IgM antibodies at different time points and subjected to flow cytometric analysis. The percentages of cells expressing cell surface IgM are indicated. (b) Murine 70Z/3 cells were treated with goat anti-IgM antibodies (5 *μ*g/mL) for different times; cell surface IgM were monitored by APC labeled anti-IgM antibodies. The percentages of cells expressing cell surface IgM are indicated. Unstained samples were used as negative control. (c) Immunofluorescence analysis. For untreated cells, EU12 *μ*HC^+^, Daudi, or Ramos cells were fixed and stained with Cy5 labeled anti-IgM antibodies. For anti-IgM antibody treated cells, EU12 *μ*HC^+^, Daudi, and Ramos cells were treated with Cy5 labeled anti-IgM (2 *μ*g/mL) for 30 min. Cells were then fixed and visualized on glass slides with fluorescence microscopy. (d) Human EU12 *μ*HC^+^ or Daudi cells were treated with anti-IgM antibodies (2 *μ*g/mL) for 24 hours and cell surface HLA and CD86 levels were analyzed by flow cytometry.

Our previous studies showed that treatment of EU12 *μ*HC^+^ cells with goat anti-human IgM antibody F(ab′)_2_ fragments resulted in complete internalization of surface BCRs [21]. Here, we compared anti-IgM antibody-mediated BCR internalization in the EU12 *μ*HC^+^ immature B cell, versus those in Daudi and Ramos mature B cells. In the EU12 *μ*HC^+^ cells, crosslinking BCRs with anti-IgM antibodies resulted in quick BCR internalization, which can be seen as soon as 1 min (Figure 1(a)). After 30 min, almost all the immature cells lost their cell surface BCRs (Figure 1(a)). In contrast, treatment of Daudi and Ramos cells with the same amount of anti-IgM antibodies only resulted in partial internalization of BCRs (Figure 1(a)). After 4 h, about 96% of Daudi cells and 78% of Ramos cells still expressed their cell surface BCRs (Figure 1(a)).

To determine if complete internalization of cell surface BCRs is a general mechanism for early B lineage cells, we analyzed a panel of murine early B lineage cells and found that crosslinking cell surface BCRs also induced complete internalization of cell surface BCRs in 70Z/3 cells (Figure 1(b)). 

To further verify these findings, the three lines of cells were treated with or without Cy5 labeled F(ab′)_2_ anti-human IgM antibody fragments (2 *μ*g/mL) at 37°C for 30 min, and cell surface BCR expression was visualized under immunofluorescence microscopy. In untreated cells, the labeled BCRs were distributed on the cell surface. After treatment, all the Cy5 labeled anti-IgM antibodies were internalized into the EU12 *μ*HC^+^ cells. For Daudi and Ramos cells, a large fraction of Cy5 labeled anti-IgM antibodies still remained on the cell surface under the same treatment (Figure 1(c)). These results show that the EU12 *μ*HC^+^ immature B cells are highly sensitive to BCR crosslinking and completely internalize their cell surface BCRs, which are different from that in the mature B cells.

Moreover, after treatment of EU12 *μ*HC^+^ cells or Daudi cells with anti-IgM antibodies (2 *μ*g) for 24 hours, we analyzed cell surface HLA and CD86 expression as indicators for B cell activation. Upon anti-IgM antibody treatment, Daudi cells have elevated HLA and CD86 expression. In contrast, the expression levels of HLA and CD86 are not changed in EU12 *μ*HC^+^ cells upon anti-IgM antibody treatment (Figure 1(d)). These results indicated that crosslinking cell surface BCRs activated different signaling in the EU12 *μ*HC^+^ cells or in Daudi cells.

### 3.2. Complete Internalization of BCRs in the EU12 *μ*HC^+^ Cells Reduces Ca^2+^ Influx upon Restimulation

**Figure 2:**
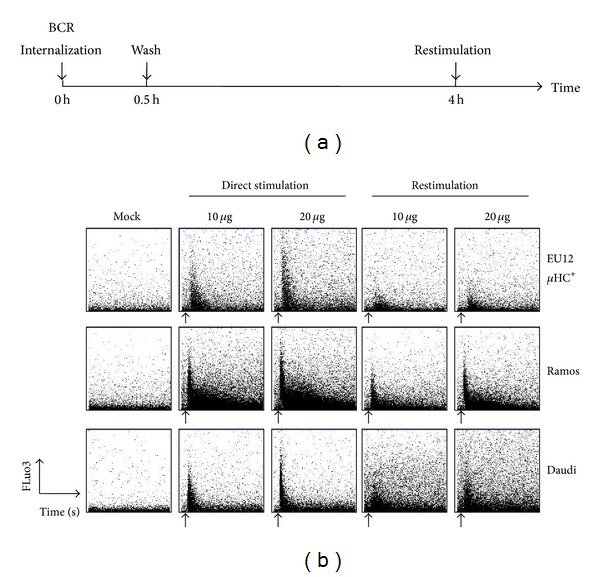
BCR internalization reduces Ca^2+^ influx upon restimulation. (a) Schematic representation of different stimulation conditions for EU12 *μ*HC^+^, Daudi, and Ramos cells with goat anti-IgM antibody F(ab′)_2_ fragments. (b) EU12 *μ*HC^+^, Daudi, and Ramos cells were prepared as outlined in (a). EU12 *μ*HC^+^, Daudi, and Ramos cells were either directly stimulated with goat F(ab′)_2_ anti-IgM antibodies (10 *μ*g/mL or 20 *μ*g/mL) or pretreated with goat anti-IgM antibody F(ab′)_2_ fragments (2 *μ*g/mL) for 30 min to internalize BCRs, washed to remove excessive anti-IgM antibodies, recovered for 4 h, and then restimulated with goat anti-IgM antibody F(ab′)_2_ fragments (10 *μ*g/mL or 20 *μ*g/mL). Baseline fluorescence was established with the mock experiments without addition of stimulatory antibodies. Arrows indicate the time points when antibodies were added. Results shown are representative of three independent experiments.

To determine the effects of complete BCR internalization on subsequent BCR-mediated signaling events, we compared EU12 *μ*HC^+^, Daudi, and Ramos cells for their capabilities to evoke calcium influx upon BCR stimulation before and after BCR internalization. Direct stimulation of the three lines of cells with goat anti-human IgM antibody F(ab′)_2_ fragments (10 *μ*g/mL or 20 *μ*g/mL) induced Ca^2+^ influx in a dose-dependent manner (Figure 2(b)). During the restimulation experiments, EU12 *μ*HC^+^ cells showed dramatically reduced Ca^2+^ influx compared to that during direct stimulation or those in restimulated Daudi and Ramos cells (Figures 2(a) and 2(b)). These results revealed that complete internalization of BCRs on the EU12 *μ*HC^+^ cells reduced Ca^2+^ influx during BCR restimulation.

### 3.3. Complete BCR Internalization in the EU12 *μ*HC^+^ Cells Specifically Changes BCR-Mediated Downstream Signaling Events

**Figure 3:**
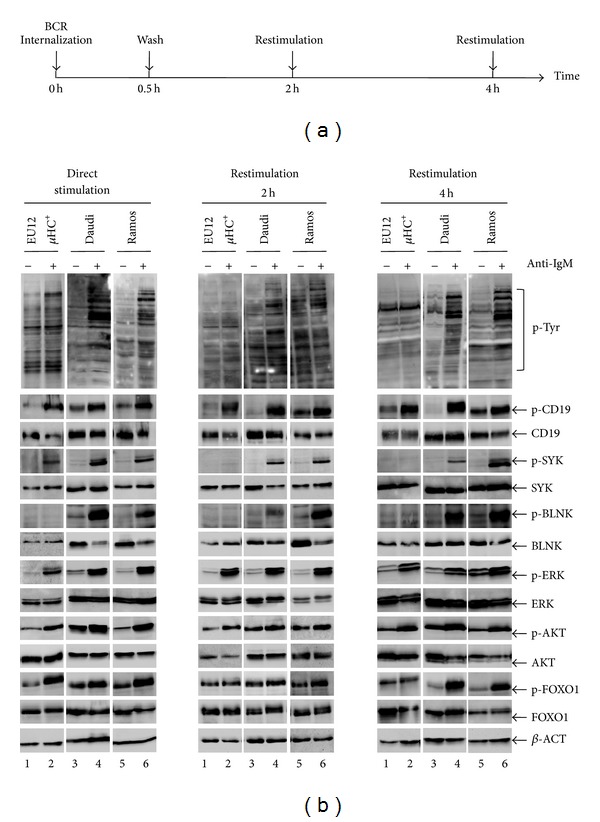
Internalization of BCR changes downstream signaling events upon restimulation. (a) Schematic representation of different treatment for EU12 *μ*HC^+^, Daudi, or Ramos cells with goat anti-IgM antibody F(ab′)_2_ fragments. (b) EU12 *μ*HC^+^, Daudi, and Ramos cells were directly stimulated with goat F(ab′)_2_ anti-IgM antibodies (10 *μ*g/mL or 20 *μ*g/mL) or pretreated with goat anti-IgM antibody F(ab′)_2_ fragments (2 *μ*g/mL) for 30 min to internalize BCRs, washed to remove excessive anti-IgM antibodies, recovered for 2 h or 4 h, and then restimulated with goat anti-IgM antibody F(ab′)_2_ fragments (10 *μ*g/mL). BCR-mediated downstream signaling events were monitored by Western blot analyses using 4G10 (p-Tyr) antibodies or antibodies specific to phosphorylated CD19 (p-CD19), phosphorylated BLNK (p-BLNK), phosphorylated ERK (p-ERK), phosphorylated AKT (p-AKT), or phosphorylated FOXO1/3a (p-FOXO1/3a). Membranes were stripped and reprobed with antibodies against CD19, Lyn, SYK, BLNK, ERK, AKT, FOXO1, and *β*-ACTIN as controls.

To further determine if BCR internalization affects BCR-mediated downstream signaling events, we performed Western blot analyses to determine the phosphorylation status of different BCR signaling molecules. As described before, the three lines of cells were either directly stimulated with goat anti-human IgM antibody F(ab′)_2_ fragments (10 *μ*g/mL) or pretreated with goat anti-human IgM antibody F(ab′)_2_ fragments (2 *μ*g/mL) at 37°C for 30 min to first internalize cell surface BCRs and then rechallenge them at 2 h or 4 h with goat anti-human IgM antibody F(ab′)_2_ fragments (10 *μ*g/mL). With direct stimulation, EU12 *μ*HC^+^ immature B cells exhibited similar activation of downstream signaling molecules, including CD19, SYK, ERK, AKT, and FOXO1, compared to those in mature Daudi and Ramos cells. However, BCR signaling induced BLNK phosphorylation was almost undetectable in the EU12 *μ*HC^+^ cells (Figure 3(b)). Restimulation of EU12 *μ*HC^+^ cells induced no apparent changes of global protein Tyrosine phosphorylation at 2 h or 4 h time points. Interestingly, restimulation of EU12 *μ*HC^+^ cells did not induce SYK and FOXO1 phosphorylation at 3 min but still induced similar levels of ERK phosphorylation. Taken together, complete internalization of BCRs in the EU12 *μ*HC^+^ cells not only attenuates BCR-mediated signaling events, but also specifically changes the downstream signaling events with reduced SYK activation but with sustained ERK activation.

### 3.4. Delayed SYK Activation and Sustained ERK Phosphorylation after BCR Internalization 

**Figure 4:**
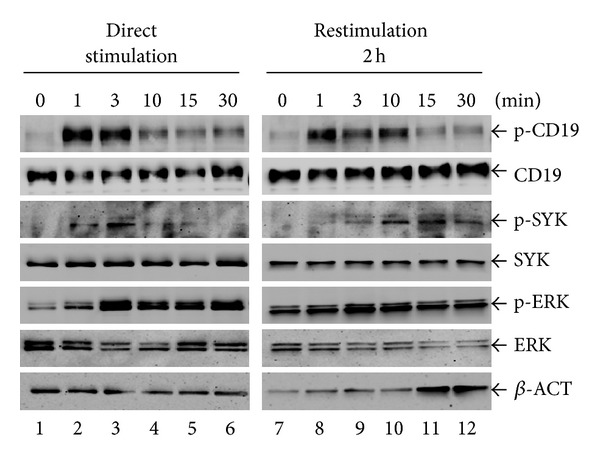
Internalization of BCR results in delayed activation of SYK and sustained activation of ERK1/2 in EU12 *μ*HC^+^ cells upon restimulation. EU12 *μ*HC^+^ cells were directly stimulated with goat anti-IgM antibody F(ab′)_2_ fragments (10 *μ*g/mL) or pretreated with goat anti-IgM antibody F(ab′)_2_ fragments (2 *μ*g/mL) for 30 min, washed, recovered, and restimulated upon goat anti-IgM antibody F(ab′)_2_ fragments (10 *μ*g/mL). Cells were collected at 0, 1, 3, 10, 15, and 30 min and analyzed for CD19, SYK, and ERK phosphorylation. The membranes were stripped and blotted with anti-CD19, anti-SYK, anti-ERK, or anti-*β*-ACTIN antibodies as controls.

In mature B cells, engagement of surface BCRs activates SYK. Activated SYK phosphorylates BLNK, thereby leading to the formation of BCR signalosome to fully activate the downstream PI3K, mitogen-activated protein kinase (MAPK), and NF-*κ*B pathways [26]. In our study, however, after complete internalization of BCRs in EU12 *μ*HC^+^ cells, restimulation with anti-IgM antibody F(ab′)_2_ fragments (10 *μ*g/mL) did not activate SYK at 3 min. To further determine the activation status of SYK in the EU12 *μ*HC^+^ cells during restimulation process, we analyzed SYK phosphorylation at different time points after either direct stimulation or restimulation. During direct stimulation, SYK phosphorylation can be detected as soon as 1 min after BCR crosslinking. It reached the peak level of phosphorylation at 3 min and then diminished after 10 min. In contrast, in restimulated EU12 *μ*HC^+^ cells, SYK phosphorylation was barely detectable at 1 or 3 min, increased at 10 and 15 min, and lasted till 30 min (Figure 4). The level of CD19 phosphorylation was also reduced during restimulation. Surprisingly, ERK phosphorylation was sustained after 30 min even after all the cells lost their cell surface BCRs during the direct stimulation (Figure 4, lane 6). The level of ERK phosphorylation was further enhanced during restimulation after 2 h (Figure 4, lanes 8 and 9). Similar results were obtained using either goat F(ab′)_2_ anti-IgM antibody fragments or monoclonal anti-human IgM antibodies. These results support that complete internalization of BCRs in the EU12 *μ*HC^+^ immature B cells altered the downstream signaling pathways.

### 3.5. Distribution of Phosphorylated SYK and ERK in Stimulated EU12 *μ*HC^+^ Cells

**Figure 5:**
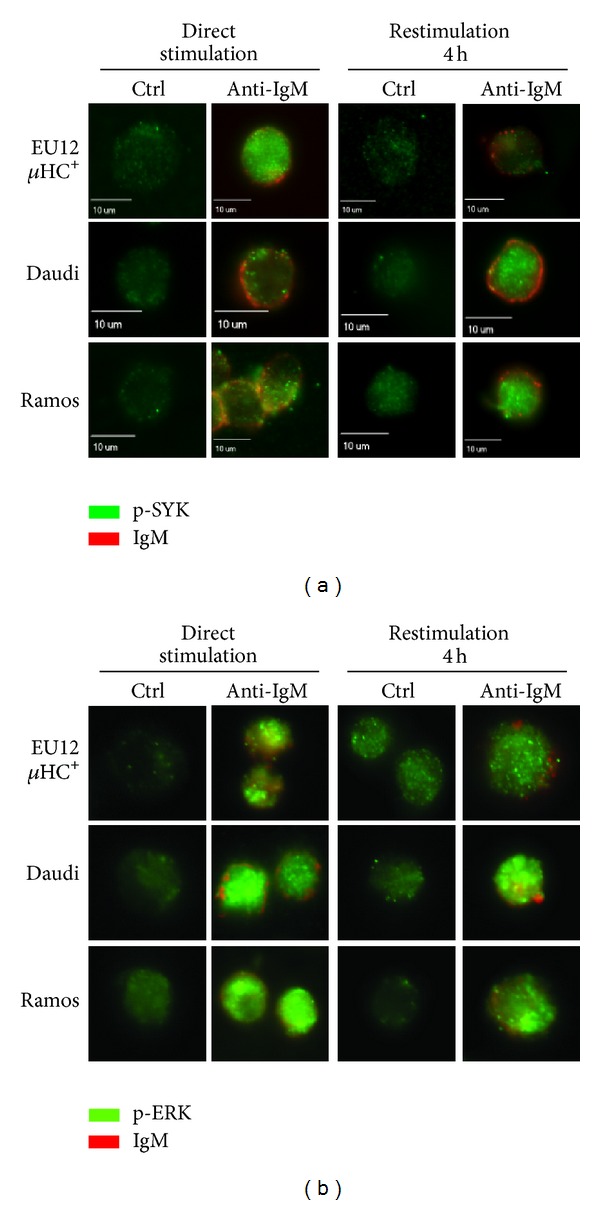
Internalization of BCR changes downstream signaling events upon restimulation. EU12 *μ*HC^+^, Daudi, and Ramos cells were directly stimulated with Cy5 labeled anti-IgM antibodies (10 *μ*g/mL) for 3 min or pretreated with goat anti-IgM antibody F(ab′)_2_ fragments (2 *μ*g/mL) for 30 min, washed, recovered for 4 h, and restimulated with Cy5 labeled anti-IgM antibodies (10 *μ*g/mL) for 3 min. Fixed and permeabilized cells were analyzed by immunofluorescence microscopy for phosphorylated SYK (a) and ERK1/2 (b). Scale bar, 10 *μ*m. Results shown are representatives from three independent experiments with more than 150 cells analyzed.

Next, we performed immunofluorescence staining to visualize phosphorylated SYK and ERK proteins in these three lines of cells upon direct stimulation or restimulation. In directly stimulated EU12 *μ*HC^+^, Daudi, or Ramos cells, crosslinking BCR with Cy5 labeled F(ab′)_2_ anti-IgM antibodies (10 *μ*g/mL) induced SYK and ERK phosphorylation (Figures 5(a) and 5(b), left panels). In restimulated EU12 *μ*HC^+^ cells, SYK phosphorylation was almost undetectable. Interestingly, phosphorylated ERK could be detected inside of the EU12 *μ*HC^+^ cells even 4 h after the first stimulation and was further enhanced by restimulation with anti-IgM antibodies (Figures 5(a) and 5(b), right panels). These results are consistent with the Western blot results, which indicate that complete internalization of BCRs in the EU12 *μ*HC^+^ cells specifically changes BCR-mediated downstream signaling with sustained ERK activation.

## 4. Discussion

BCRs expressed on immature B cells and mature B cells have the same components; however, upon stimulation, they transduce different signals, which result in different outcomes [27]. For immature B cells, engagement of BCR induces receptor editing, clonal deletion, or anergy; for mature B cells, stimulation of BCR induces cell activation and proliferation. Understanding how the same BCRs can generate different signals is a long standing question in the immunology research field. 

We are currently using the EU12 *μ*HC^+^ cells as an experimental model system to study the molecular regulation of *V*_*H*_ replacement. The EU12 *μ*HC^+^ cells phenotypically resemble human bone marrow immature B cells [20]. We notice that the EU12 *μ*HC^+^ cells are highly sensitive to BCR crosslinking that internalizes their cell surface BCRs [21]. Treatment of EU12 *μ*HC^+^ cells with low concentration of goat anti-human IgM antibody F(ab′)_2_ fragments (2 *μ*g/mL) resulted in quick internalization of cell surface BCRs, which could be seen as soon as 1 min after adding the antibodies. After 30 min, almost all the EU12 *μ*HC^+^ cells lost their cell surface BCRs. The complete BCR internalization observed with the EU12 *μ*HC^+^ cells was different from the partial BCR internalization that occurred in mature B lineage Daudi or Ramos cells. Under the same stimulations, the majority of the Daudi and Ramos cells still have their cell surface BCRs, although the levels of cell surface BCRs are slightly reduced. Complete internalization of cell surface BCRs upon BCR crosslinking seems to be a general phenomenon for early B linage cells, because upon anti-IgM antibody treatment, murine 70Z/3 cells also completely internalize cell surface BCRs. Currently, it is not clear why EU12 *μ*HC^+^ cells and 70Z/3 cells quickly and completely internalize their cell surface BCRs upon anti-IgM treatment. It has been shown that another murine B lineage cell line, WEHI231, which represents murine immature B cells, did not internalize cell surface BCRs upon anti-IgM antibody treatment [28]. It will be very interesting to further compare and contrast BCR signaling mediated events in these early B lineage cell lines to identify the responsive factors that control BCR internalization. 

Treatment of EU12 *μ*HC^+^ cells or Daudi cells with the same concentration of anti-IgM antibodies resulted in different activation of HLA and CD86. We speculate that internalization of cell surface BCRs will impact the downstream signaling events. Detailed analysis showed that restimulation of EU12 *μ*HC^+^ cells after complete internalization of cell surface BCRs showed dramatic reduction of BCR induced Ca^++^ influx. Furthermore, after BCR internalization, restimulated EU12 *μ*HC^+^ cells failed to induce global protein tyrosine phosphorylation even with higher concentration of the stimulating anti-IgM antibodies at 2 h or 4 h. Under the same treatment, Ca^++^ influx in Daudi or Ramos cells was also attenuated, but restimulation still induced strong protein tyrosine phosphorylation. Such results can be easily explained as that reducing the cell surface BCR expression levels attenuates BCR-mediated signaling events. 

More specifically, after complete internalization of BCRs, restimulation of EU12 *μ*HC^+^ cells resulted in a delayed SYK phosphorylation and reduced CD19 and FOXO1 phosphorylation. Such changes are consistent with the low level of BCR expression on the EU12 cell surface. However, anti-IgM antibody-mediated activation of ERK in the EU12 *μ*HC^+^ cells was not affected by complete internalization of BCRs. on the contrary, sustained ERK phosphorylation was detected in the EU12 *μ*HC^+^ cells even after they lost cell surface BCRs. Restimulation of EU12 *μ*HC^+^ cells induced a similar wave of ERK activation. These results show that internalization of BCR in the EU12 *μ*HC^+^ cells specifically changed BCR-mediated downstream signaling events with sustained ERK activation but reduced SYK, CD19, and FOXO1 phosphorylation. Such changes may be due to the differential activation thresholds of individual signaling factors. For ERK, the activation threshold may be very low. With minimal levels of cell surface BCR stimulation, ERK can be phosphorylated to activate the downstream signaling events. On the other hand, the sustained ERK activation after BCR internalization suggested that the internalized BCRs within the endosomes might still be able to activate ERK. Such low threshold of ERK activation and sustained ERK activation might be critical for immature B cells for induction of receptor editing and negative selection with even low affinity self-antigens. However, after internalization of the majority of the cell surface BCRs, SYK can no longer be quickly and fully activated in the EU12 *μ*HC^+^ immature B cells after restimulation. Given the important roles of SYK in BCR-mediated early signaling events in mature B cells, delayed and attenuated SYK activation might affect the formation of the signalosome to fully activate Ca^++^ influx and the downstream PI3K and NF-*κ*B pathways. Indeed, we observed reduced Ca^++^ influx and reduced CD19 and FOXO1 phosphorylation upon restimulation of EU12 *μ*HC^+^ cells. Recent studies have shown that ERK activation is involved in light chain receptor editing process [29]. Correlating with the sustained activation of ERK in the EU12 *μ*HC^+^ cells, we suspect that ERK activation is important for induction of *V*_*H*_ replacement in human immature B cells. 

Among the different BCR-mediated downstream signaling pathways, induction of Ca^++^ influx, activation of the PI3K, and NF-*κ*B pathways are always considered to be important for B cell activation and proliferation. Here, our results show that complete internalization of BCRs in the EU12 *μ*HC^+^ cells specifically alters the BCR downstream signaling events with sustained activation of ERK but attenuated Ca^++^ influx and CD19 and FOXO1 phosphorylation, which are different from that in mature B cells. We speculate that such changes in the EU12 *μ*HC^+^ immature B cells may prevent B cell activation and proliferation from favoring the induction of *V*_*H*_ replacement.

## 5. Conclusions

Crosslinking BCRs on EU12 *μ*HC^+^ immature B cells completely internalizes cell surface BCRs and specifically alters the BCR downstream signaling events with sustained activation of ERK but reduced Ca^++^ influx and SYK, CD19, and FOXO1 phosphorylation. Such changes may favor the induction of *V*_*H*_ replacement rather than cell activation and proliferation in the EU12 *μ*HC^+^ immature B cells.
